# Laparoscopic Pyeloplasty for Ureteropelvic Junctions Obstruction in Adults: 6 Years' Experience in One Center

**DOI:** 10.1155/2017/6743512

**Published:** 2017-06-11

**Authors:** Rikke Søgaard Tolstrup, Marie Thue Pank, Lotte Sander, Torben Dørflinger

**Affiliations:** Department of Urology, Aalborg University Hospital, Aalborg, Denmark

## Abstract

**Objective:**

Ureteropelvic junction obstruction (UPJO) is a common cause of symptomatic ureteral obstruction. The aim of this study is to assess the outcome of laparoscopic pyeloplasty in patients with UPJO.

**Patients and Methods:**

Medical reports of 47 UPJO patients treated with laparoscopic pyeloplasty were retrospectively analysed. All patients were recruited from our center in the period 2004–2011.

**Results:**

We evaluated 47 patients. Mean age was 36 years and mean hospital stay 3.6 days. 42 (79%) of the patients had pain and 46 (98%) were diagnosed with hydronephrosis. 19 patients (40%) had a renal function below 40% of the affected kidney and 49% had impaired renal scan drainage. Postoperative significant improvement in pain score and renal scan drainage was found in 92% and 47% of the patients, respectively. Improvement of renal function > 10% was found in 11 patients (23%); the function remained stable in 31 patients (66%) and deteriorated > 10% in 5 patients (11%). We found no correlation between sex or age and the outcome.

**Conclusion:**

Laparoscopic pyeloplasty for UPJO leads to relief of pain and preserved or improved renal function in the majority of the patients. Overall laparoscopic pyeloplasty is an efficient treatment for UPJO.

## 1. Introduction

Ureteropelvic junction obstruction (UPJO) either idiopathic, iatrogenic, or due to compression of aberrant vessels is a common urological problem. Untreated the disease can cause renal failure, urinary tract infection, urolithiasis, and other symptoms as pain [1].

The primary gold of intervention is to preserve or improve renal function and relieve symptoms. Surgical reconstruction is the golden standard for treatment and there are several options for surgical intervention [2].

### 1.1. Endourologic Management

Today the endourologic management is mostly performed as a retrograde ureteroscopic endopyelotomy. However, the procedure may be performed as percutaneous antegrade one either as a endopyelotomy or endopyeloplasty.

The procedure is performed with a lateral incision through the obstructing proximal ureter using a cold knife or the holmium laser. Alternatively the obstruction can be dilated with a cautery wire balloon [3].

### 1.2. Pyeloplasty

This surgical management can be performed as open surgery, laparoscopic or robot-assisted. The technique is performed by dissecting the part of the ureter and renal pelvis with the obstruction, then spatulating the ureter, and making an anastomosis to the renal pelvis.

**Figure 1:**
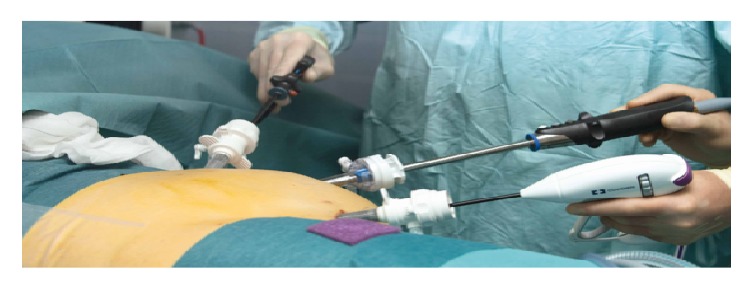
Laparoscopic pyeloplasty

Laparoscopic standard approach is transperitoneal, but the procedure can also be performed by an retroperitoneal approach, anterior extraperitoneal approach, laparoendoscopic single-site surgery approach, or robotic-assisted approach. The most widely used method is the Anderson-Hynes pyeloplasty or one of the nondismembered methods such as Foley Y-V plasty. Methods like Culp-DeWeerd spiral flap pyeloplasty, Scardino-Prince Vertical Flap, Davis intubated ureterotomy, and ureterocalicostomy can also be used [3].  Figure 1 shows the laparoscopic approach.

### 1.3. Nephrectomy

This treatment option is rarely the procedure of choice and is only used when the affected kidney is nonfunctioning, the patient has symptoms, and other treatment options are not preferred [3].

Pyeloplasty is currently the standard treatment for most cases of UPJO. The success rate with endourologic techniques is lower and has proven not to be comparable with those of pyeloplasty.

Traditionally, the surgical procedure was performed as an open pyeloplasty, but since the development of minimal invasive surgical techniques, the standard is now to perform the procedure laparoscopically.

The European Association of Urology recommended in their guidelines that standard option of treatment should be pyeloplasty performed by a laparoscopic approach [4].

In 2015 a total of 120 pyeloplasty cases, primary as minimally invasive surgery, were performed in Denmark.

The aim of this study is to assess the subjective symptoms and objective outcome in patients with UPJO undergoing a laparoscopic pyeloplasty in our center.

## 2. Patients and Methods

The study was performed as a retrospective analysis of medical reports of patients with UPJO treated with laparoscopic pyeloplasty, using the Anderson-Hynes dismembered technique or a nondismembered technique as a Y-V or flap-plasty. All medical reports were collected from our center: The Department of Urology, Aalborg University Hospital, between 2004 and 2011.

All patients with UPJO were evaluated, and only patients who had undergone pyeloplasty (*n* = 56) were included in this study. Patients that had open surgery were excluded, resulting in a total of 47 patients.

The included patients all had UPJO, which was mainly diagnosed by subjective symptoms such as flank pain, chronic urinary tract infection, and urolithiasis and the diagnosis was confirmed by intravenous ureterography.

Indications for surgery were symptomatic UPJO such as flank pain, urinary tract infection, impaired renal function, and/or decline in renal function over time monitored on a diuretic renal scan.

All patients who preoperatively had symptoms underwent surgery with cystoscopy insertion of a double-J stent. If the stent showed a beneficial effect on the symptoms, the patients were considered for operation.

All the patients had laparoscopic surgery with pyeloplasty either as conventional laparoscopy or as a robot-assisted procedure. The surgical technique of choice was surgeon dependent; however in general all patients with aberrant vessel had the pyeloplasty by the Anderson-Hynes technique. The remaining patients with no aberrant vessels had either Anderson-Hynes, Y-V, or flap-plasty, depending on size and ethology of the obstruction and the surgeon.

During surgery a double-J stent was routinely inserted in all patients; the stent was removed at follow-up.

At follow-up the patients' subjective symptoms were evaluated as well as the objective outcome, which were monitored by ureterography and renal scans.

## 3. Results

The medical reports of a total of 56 patients who underwent pyeloplasty were examined; 9 patients were excluded because they had open procedure, leaving 47 patients available for evaluation, 24 females and 23 males. All 47 patients were characterised by gender, age, and mean hospital stay (Table 1). At surgery, the patient's age ranged from 15 to 73 years; mean age was 36 years.

The mean hospital stay was 3.6 days, ranging from 2 to 8 days.

**Figure 2:**
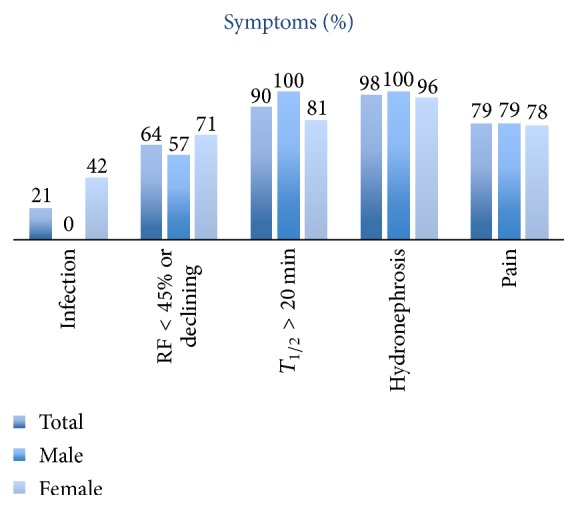
Preoperative symptoms and objective findings.

In order to assess the outcome after pyeloplasty, postoperative symptoms and objective findings were identified (Figure 2).

79% of the patients had subjective symptoms before operation dominated by flank pain. 98% patients were diagnosed with hydronephrosis in preoperative imaging. 64% of the patients had renal function blow 45% of the affected kidney at renography, while a function below 40% was seen in 40% of the patients. Impaired renal scan drainage was found in 49% of the patients.

Mean days until follow-up were 45,5 days with a range from 18 to 151 days.

**Figure 3:**
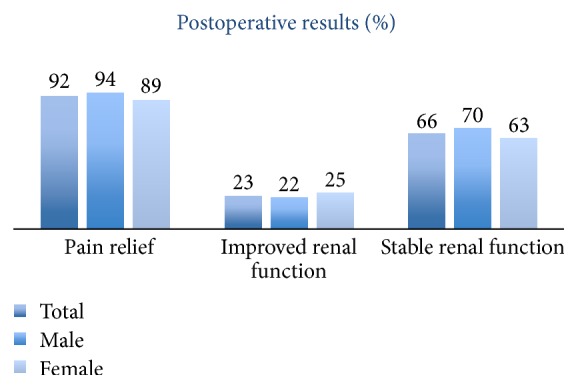
Postoperative results.

The results after operation are presented in Figure 3.

Postoperative significant improvement in pain score and renal scan drainage was found in 92% and 47% of the patients, respectively. Postoperative imaging showed improvement in 65% of the patients.

Improved renal function more than 10% was seen in 11 patients (23%); the function remained stable in 31 patients (66%) and deteriorated > 10% in 5 patients (11%).

We found no correlation between sex or age and the outcome of the operation.

One patient required repeat pyeloplasty due to reobstruction. Furthermore one patient underwent a reoperation due to severe postoperative pain.

## 4. Discussion

In this study we show that laparoscopic pyeloplasty is an efficient treatment for UPJO, with a significant improvement in pain and renal scan drainage in 92% and 47% of the patients, respectively.

89% of the patients had stabilized their renal function or even improved it with more than 10%. The result must be taken into consideration that the study population was quite small with only 47 patients.

This study was performed as a retrospective analysis based on medical reports, which has its limitations. There were no standardizations on subjective and objective symptoms. The patients did not follow a standard programme for presurgery examination or postoperative follow-up.

The use of validated questionnaires for assessment of subjective symptoms, as pain, would have strengthened our study.

The urography was performed and described by different radiologists and there was no standard or measurement for the degree of hydronephrosis.

In our study population there was no differentiation between patients who had conventional laparoscopic procedure or robot-assisted one (Da Vinci robot system). Furthermore there was no differentiation in the etiology causing the UPJO and the surgical technique used.

Our results are equal to a recent meta-analysis by Wang et al. who shows an overall effect on laparoscopic pyeloplasty on 88% of the patients [5].

Recent studies have shown similar results and no statistical significant difference in overall success rates comparing traditionally open surgery technique with minimal invasive surgery.

Several studies have demonstrated that adults with UPJO treated with pyeloplasty as minimally invasive surgery have a lower risk of complications, transfusions, prolonged hospital stay, and cosmetic outcome and a general lower morbidity compared to patients that had open surgery [6, 7].

In contrast, pyeloplasty requires more significant skills by the operator to do the intercorporeal knotting witch prolonged the operation time [8].

A systematic review and meta-analysis of 12 studies by Wang et al. shows that robot-assisted pyeloplasty achieves equivalent results compared to conventionally laparoscopic pyeloplasty, but the robot-assisted procedure has several advantages by shorter suturing-time and shorter length of hospital stay. Furthermore, the robot-assisted procedure has better ergonomics for the surgeon, which improves the success rate of suturing the anastomosis due to more degrees of freedom for movements [5, 9].

In 2008, the robot-assisted procedures were introduced in our center with the Da Vinci system, and the numbers of robot-assisted minimal invasive procedures have only increased. The newest report from Sociatas Urologica Denica 2015 shows that 87% of the pyeloplasty in Denmark are performed with robot assistance [10].

More studies within this field are needed to compare the success rate of our laparoscopic procedures performed traditionally versus robot-assisted procedures. Further studies should be performed under standardized conditions with better measurements of subjective symptoms and objective outcome, simultaneous with a longer follow-up, to evaluate long term outcome.

## 5. Conclusion

Laparoscopic pyeloplasty for UPJO leads to relief of subjective symptoms such as pain in the majority of the patients. Regarding objective outcomes the renal function was mainly preserved or improved. Renal scan drainage showed significant improvement. The success rates are comparable with the results from other centers.

Overall laparoscopic pyeloplasty is an efficient treatment for UPJO.

## Figures and Tables

**Table 1:** Characteristics of patients with UPJO.

Characteristic	Number (range)
Number of patients	47
(i) Male	23
(ii) Female	24
Age, yr.: mean (range)	36 (15–73)
(i) Male	32 (15–68)
(ii) Female	40 (18–73)
Days in hospital: mean (range)	3,6 (2–8)
(i) Male	3,7 (2–8)
(ii) Female	3,4 (2–7)
